# Editorial: The molecular mechanisms and therapeutic targets of atherosclerosis

**DOI:** 10.3389/fcvm.2022.1127693

**Published:** 2023-01-10

**Authors:** Roberta Gualtierotti, Massimiliano Ruscica

**Affiliations:** ^1^Fondazione Istituti di Ricovero e Cura a Carattere Scientifico (IRCCS) Ca' Granda Ospedale Maggiore Policlinico, S.C. Medicina - Emostasi e Trombosi, Milan, Italy; ^2^Department of Pathophysiology and Transplantation, Università degli Studi di Milano, Milan, Italy; ^3^Department of Pharmacological and Biomolecular Sciences, Università degli Studi di Milano, Milan, Italy

**Keywords:** proprotein convertase subtilisin/kexin type 9 (PCSK9), brain derived neurotrophic factor (BDNF), rheumatoid arthritis, inflammation, extracellular vesicles

While many studies have provided considerable insight into the mechanisms leading to atheroma development, the burden of atherosclerotic cardiovascular diseases (ASCVD) spans the globe. Despite great advancements in tackling ASCVD with the implementation of traditional risk factor-based preventive measures and evidence-based treatment recommendations, risk stratification models still fail to accurately assess cardiovascular (CV) risk. Besides traditional risk factors (e.g., low-density lipoprotein cholesterol - LDL-c), many non-traditional drivers have to be considered (e.g., mood disorders or environmental stress) (1). Thus, it becomes of interest to understand the role of extracellular vesicles (EVs) either as diagnostic and prognostic tools or as mechanistic mediators of ASCVD (2). Virtually released by all cell types, EVs are nanosized vesicles with a lipid bilayer containing biological information (e.g., DNA, RNA or proteins) that can be transferred to distal tissues to exert their actions. EVs are present in plasma, where they are derived primarily from erythrocytes, platelets, endothelial, and immune cells. Therefore to use EVs as diagnostic markers, it is mandatory to purify them otherwise they would be obscured by the large quantity of proteins present in the fluid (3).

Accruing evidence has revealed the ability of small non-coding RNAs (18–22 nucleotides), including microRNAs (miRNAs), to regulate cellular and molecular processes during all stages of atherosclerosis (i.e., cell invasion, growth and survival, cellular uptake and efflux of lipids, expression and release of pro- and anti-inflammatory intermediaries, and proteolytic balance) (4). miRNA can control mRNA/protein expression post-transcriptionally through inhibition of translation or promotion of targeted messenger degradation. miRNAs are considered, by many authors, to be exclusively released within and protected by EVs. Being made out of single-strand RNA, miRNAs are instable and rapidly degraded in blood by RNAses, unless specifically protected. However, the functional role of EV-derived-miRNA has been recently called into question. EVs can only carry a low number of miRNA molecules that are too few to mediate a significant biological effect in recipient cells (5, 6). However, in associative studies that cannot imply causality, it seems irrelevant whether circulating miRNAs transmit biological information to their target cells (7). In this Research Topic, two out of four manuscripts dealt with the field of EV-derived miRNAs as possible biomarkers of CV risk linked to obesity (Macchi et al.) and depression (Amadio et al.). Macchi et al. epidemiologically demonstrated an association between EV-derived miRNAs and proprotein convertase subtilisin/kexin type 9 (PCSK9), a player in atheroma development (8). In a cohort of 936 individuals with obesity, PCSK9 was significantly associated with five EV-derived miRNAs (hsa-miRNA−362–5p, −150, −1,244, −520b−3p, −638), with LDL receptor, toll-like receptor 4 and estrogen receptor 1 being the predicted gene targets. However, the molecular determinant of this liaison needs to be unraveled (9).

In the framework of non-classical CV risk factors, the association between obesity and depression cannot be underestimated, since depression increases markedly the risk to develop CV complications in individuals with obesity. Brain derived neurotrophic factor (BDNF) is a neurotrophin, playing a pivotal role in several physiological processes and pathological conditions, including neuroplasticity, energy homeostasis, and CV function (10, 11). In 743 individuals with obesity, Amadio et al. found that circulating BDNF was linked to EV-derived miRNAs related to atherosclerosis and thrombosis. Network-analysis identified at least 18 genes targeted by these miRNAs, seven of which were involved in both depression and ASCVD risk.

Moving to mechanisms leading to the progression of atherosclerosis, it is imperative to study the role played by inflammation, which orchestrates each stage of the life cycle of atherosclerotic plaques (12). CC-motif chemokine ligand 2 (CCL2) is a key regulator of monocyte trafficking, as it represents one of the strongest recruitment signals for monocytes to sites of inflammation (13). In *apoE*^−/−^ mice, Tang et al. and Amadio et al. demonstrated that the deletion of Y-box binding protein 1 (YB1) in vascular smooth muscle cells reduced the expression of CCL2 by promoting glucocorticoid receptor-mediated mRNA decay and YB1 inactivation. YB1 is a ubiquitously expressed member of the cold shock protein family that binds to RNA and DNA to regulate transcription, RNA splicing, and translation. A direct link between YB1 and atherogenesis was previously demonstrated (14).

It is now clear that the crosstalk between coagulation and inflammation is relevant in the inflammatory joint diseases (e.g., rheumatoid arthritis - RA) (15). In this disease, systemic inflammation can be reduced effectively by using disease-modifying anti-rheumatic drugs (DMARDs), and the European Alliance of Associations for Rheumatology (EULAR) recommendations for CV risk management in RA emphasize the importance of reducing inflammation in RA regardless of the type of drug (16). In this Research Topic, Giachi et al. provided an update on the protective effects of DMARDs on CV risk in RA patients, offering an overview on the different steps that lead to accelerated atherosclerosis and highlighting the interaction between inflammation, thrombosis and increased CV burden. Authors discussed the protective effects on CV risk of biological DMARDs such as inhibitors of interleukin (IL)-6, IL-1, and tumor necrosis factor-alpha, as well as the controversial safety of JAK inhibitors, although, in this case, an overall protective effect was demonstrated in patients with active, and uncontrolled disease (17). Despite detrimental effects on lipid profile and disease-specific CV risk factors, the evidence that DMARDs have a protective CV effect confirms the role of inflammation in the pathogenesis of atherosclerosis in patients suffering RA and highlights the importance of counteracting this burden to reduce both disease activity and CV risk.

Giachi et al. suggested a strict yearly CV assessment, employing traditional CV risk algorithms with a correction factor to account for RA-specific risk, following the EULAR recommendations (16). Extensive lifestyle advice and management of conditions such as dyslipidaemia or hypertension should not differ from general population, and a multidisciplinary and personalized approach is advised. A future strategy to reduce the burden of RA may rely on precision medicine algorithms that consider not only the genetic and metabolic background of each patient, but also the positive and negative effects of specific drugs on the associated comorbidities.

## Author contributions

All authors listed have made a substantial, direct, and intellectual contribution to the work and approved it for publication.
